# Supplementary material for: k-mer manifold approximation and projection for visualizing DNA sequences
Source: Genome Res. 2025 May;35(5):1234–46. doi: 10.1101/gr.279458.124 (PMC12047656; doi:10.1101/gr.279458.124)

## tSNE Plot - POU2F3_TAGACG20NCAT_Y_4

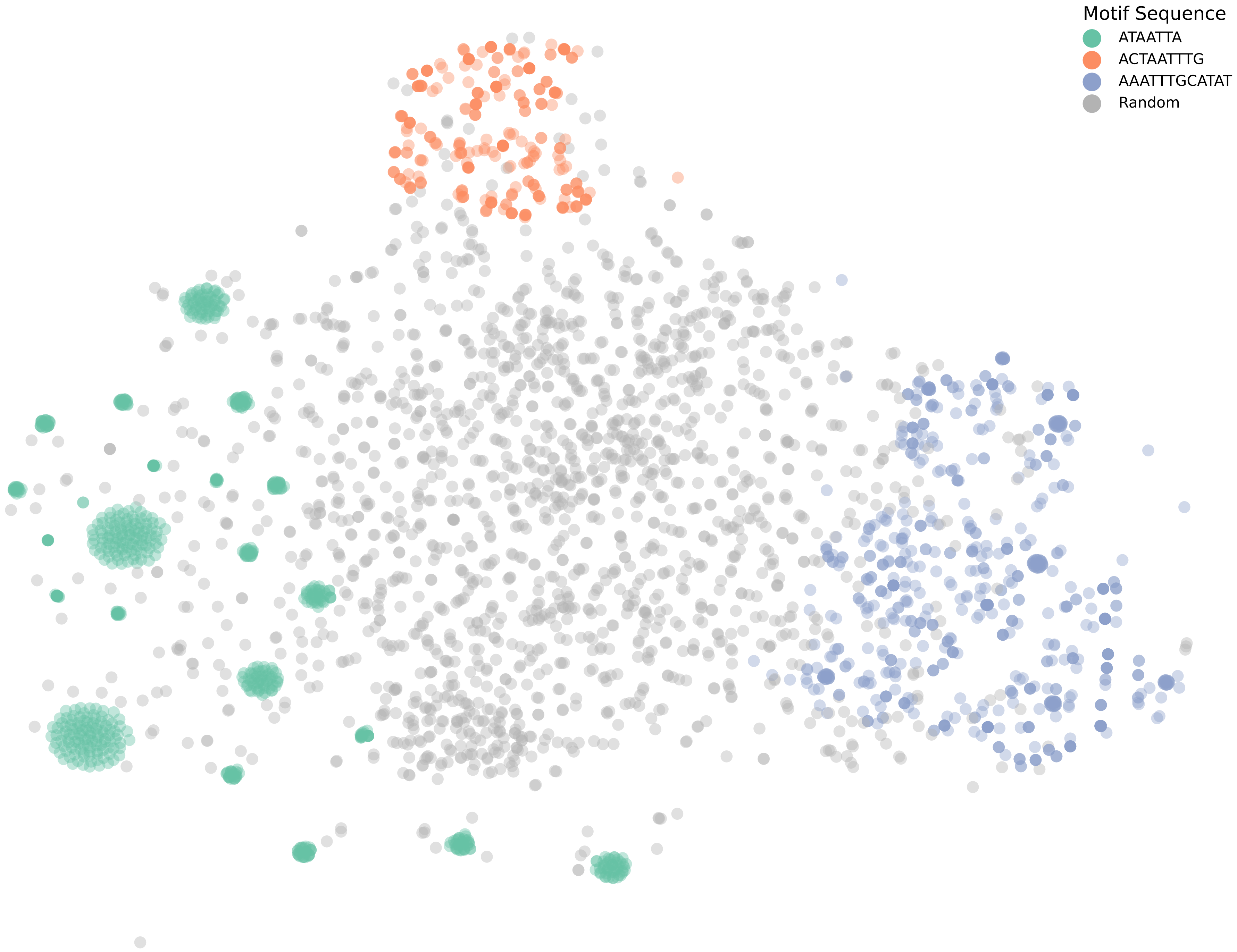

Supplement: Supplement 8 [file Supplemental_Data_1.zip › Supplemental_Data_1/POU2F3_TAGACG20NCAT_Y_4/POU2F3_TAGACG20NCAT_Y_4_tSNE.pdf]
